# Rivaroxaban versus warfarin for treatment and prevention of recurrence of venous thromboembolism in African American patients: a retrospective cohort analysis

**DOI:** 10.1186/s12959-020-00219-w

**Published:** 2020-04-08

**Authors:** Olivia S. Costa, Stanley Thompson, Veronica Ashton, Michael Palladino, Thomas J. Bunz, Craig I. Coleman

**Affiliations:** 1 Department of Pharmacy Practice, University of Connecticut School of Pharmacy, 69 North Eagleville Road, Unit 3092, Storrs, CT 06269 USA; 2 Evidence-Based Practice Center, Hartford Hospital, Hartford, CT USA; 3 TeamHealth LifePoint Group, Southaven, MS USA; 4 Real World Value and Evidence, Janssen Scientific Affairs LLC, Titusville, NJ USA; 5 Medical Affairs, Janssen Pharmaceuticals Inc., Titusville, NJ USA; 6 Department of Pharmacoepidemiology, New England Health Analytics LLC, Granby, CT USA

**Keywords:** African Americans, Anticoagulants, Rivaroxaban, Venous thromboembolism, Warfarin

## Abstract

**Background:**

African Americans are under-represented in trials evaluating oral anticoagulants for the treatment of acute venous thromboembolism (VTE). The aim of this study was to evaluate the effectiveness and safety of rivaroxaban versus warfarin for the treatment of VTE in African Americans.

**Methods:**

We utilized Optum® De-Identified Electronic Health Record data from 11/1/2012–9/30/2018. We included African Americans experiencing an acute VTE during a hospital or emergency department visit, who received rivaroxaban or warfarin as their first oral anticoagulant within 7-days of the acute VTE event and had ≥1 provider visit in the prior 12-months. Differences in baseline characteristics between cohorts were adjusted using inverse probability-of-treatment weighting based on propensity scores (standard differences < 0.10 were achieved for all covariates). Our primary endpoint was the composite of recurrent VTE or major bleeding at 6-months. Three- and 12-month timepoints were also assessed. Secondary endpoints included recurrent VTE and major bleeding as individual endpoints. Cohort risk was compared using Cox regression and reported as hazard ratios (HRs) with 95% confidence intervals (CIs).

**Results:**

We identified 2097 rivaroxaban and 2842 warfarin users with incident VTE. At 6-months, no significant differences in the composite endpoint (HR = 0.96, 95%CI = 0.75–1.24), recurrent VTE (HR = 1.02, 95%CI = 0.76–1.36) or major bleeding alone (HR = 0.93, 95%CI = 0.59–1.47) were observed between cohorts. Analysis at 3- and 12-months provided consistent findings for these endpoints.

**Conclusions:**

In African Americans experiencing an acute VTE, no significant difference in the incidence of recurrent VTE or major bleeding was observed between patients receiving rivaroxaban or warfarin.

## Background

There is ample evidence suggesting African Americans have an increased incidence of venous thromboembolism (VTE) and poorer disease outcomes compared to other racial groups [1–5]. Despite race-based differences in VTE incidence and prognosis, African Americans have been under-represented in randomized controlled trials (RCTs) evaluating non-vitamin K oral anticoagulants (NOACs) for the management of VTE [6–10]. As a result, there is a scarcity of data evaluating NOACs for the treatment and secondary prevention of VTE.

The objective of this study was to evaluate the effectiveness and safety of rivaroxaban versus warfarin in the treatment and prevention of recurrent VTE in African Americans managed in routine practice.

## Methods

We performed a retrospective cohort analysis using United States (US) Optum® De-Identified Electronic Health Record (EHR) data from November 1, 2012 to September 30, 2018 [11]. The Optum EHR database includes longitudinal patient-level medical record data for 97 million patients seen at 700 hospitals and 7000 clinics across the United States. The database includes records of prescriptions as prescribed and administered, lab results, vital signs, body measurements, diagnoses, procedures, and information derived from clinical notes using natural language processing. This database contains data on insured and uninsured patients of all ages and races to provide a representative sample of all African American patients with acute VTE in the US.

To be included in this study patients had to be African American, admitted to the hospital, emergency department or observation unit for acute deep vein thrombosis (DVT) or pulmonary embolism (PE), have received rivaroxaban or warfarin as their first oral anticoagulant (OAC) within 7-days of the acute VTE event (index date) and had at least one provider visit in the 12-months prior to the acute VTE event (baseline period). We excluded patients with another indication for OAC use (i.e. atrial fibrillation, prophylaxis after hip/knee replacement, valvular heart disease).

Our primary endpoint for this analysis was the composite of recurrent VTE or major bleeding at 3-, 6- and 12-months [12, 13]. Recurrent VTE was defined as a subsequent hospitalization with a primary International Classification of Diseases-10th Revision (or cross-walked ICD-9 to ICD-10) diagnosis code for DVT (ICD-10 = 180–182) or PE (ICD-10 = 126) [12]. Major bleeding was defined as a subsequent hospitalization for a bleeding event using the validated Cunningham algorithm [13]. This algorithm defines a hospitalization as bleeding-related based on the presence of an ICD-10 bleeding code in the primary position or the presence of select non-primary diagnosis ICD-10 codes accompanied by a billing code indicating a blood transfusion or processing of blood products for transfusion. Secondary endpoints included recurrent VTE and major bleeding as separate endpoints, as well as, intracranial hemorrhage (ICH), gastrointestinal bleeding (GIB) and genitourinary bleeding (GUB). Patients were followed for up to 12-months or until endpoint occurrence, end-of-EHR activity or through September 30, 2018 (an intent-to-treat approach).

Differences in baseline characteristics between the rivaroxaban and warfarin cohorts were adjusted for using inverse probability-of-treatment weighting (IPTW) based on propensity scores [14]. For the IPTW analysis, propensity scores (and subsequent patient weights) were estimated using generalized boosted models on the basis of 10,000 regression trees using the **‘TWANG’** package (version 1.5) and **R** statistical software version 3.4.3 (The R Project for Statistical Computing) which implements an automated, nonparametric machine learning method. The weights were derived to obtain estimates of the population average treatment effect. Variables entered in the generalized boosted modeling procedure (including demographics, comorbidities and concurrent non-anticoagulant medications) are depicted in Table 1 and were identified during the 12-month baseline period. The presence of residual differences in measured covariates following cohort weighting was assessed by calculating absolute standardized differences (< 0.1 was considered well-balanced for each variable) [14].

**Table 1:** Characteristics of the Rivaroxaban and Warfarin Intent-To-Treat Cohorts After IPTW

	Rivaroxaban*N* = 2097%	Warfarin*N* = 2842%	Absolute Standardized Difference
**Demographics**			
Age, median (25, 75% range)	50 (39, 62)	51 (40, 64)	–
Age 18–49 years	45.5	43.6	0.04
Age 50–64 years	29.6	29.2	0.01
Age 65–74 years	13.1	13.7	0.02
Age 75–79 years	4.3	4.7	0.02
Age ≥ 80 years	7.5	8.9	0.05
Female sex	56.4	56.4	0.00
Pulmonary embolism (±deep vein thrombosis)	18.6	19.1	0.01
**Comorbidities**			
Chronic obstructive pulmonary disease	8.6	9.6	0.04
Asthma	13.1	13.6	0.02
Heart Failure	5.1	5.4	0.01
Hypertension	53.5	55.2	0.03
Ischemic stroke	3.6	4.3	0.04
Diabetes	22.3	23.0	0.02
Dementia	2.6	3.3	0.04
Coronary artery disease	0.8	0.7	0.01
Carotid stenosis	0.6	0.8	0.03
Peripheral vascular disease	5.5	5.8	0.01
Myocardial infarction	5.2	5.9	0.03
Percutaneous coronary intervention	3.5	4.1	0.03
Coronary artery bypass grafting	2.4	2.8	0.02
Gastrointestinal bleed	0.3	0.5	0.02
Intracranial hemorrhage	0.0	0.3	0.07
Acute kidney injury	11.0	12.5	0.05
Other kidney injury	0.3	0.5	0.04
Inflammatory bowel disease	1.2	1.6	0.04
eGFR > 90 mL/minute	53.6	51.7	0.04
eGFR 60–89 mL/minute	30.9	30.8	0.00
eGFR 30-59 mL/minute	11.1	11.3	0.01
eGFR 15–29 mL/minute	1.9	2.7	0.06
eGFR < 15 mL/minute	1.7	2.6	0.06
eGFR unknown	0.7	0.7	0.01
Liver disease	1.6	1.6	0.00
Coagulopathy	3.1	3.5	0.02
Gastroesophageal reflux disease	18.9	18.8	0.00
Anemia	25.5	27.7	0.05
Sleep apnea	10.4	10.2	0.01
Smoking	27.4	27.4	0.00
Hemorrhoids	2.3	2.6	0.02
Alcohol abuse	0.4	0.3	0.02
Anxiety	12.6	12.2	0.01
Depression	1.6	1.8	0.02
Psychosis	1.5	1.5	0.00
BMI < 18.5 kg/m^2^	1.9	1.9	0.00
BMI 18.5–24.9 kg/m^2^	15.8	16.5	0.02
BMI 25.0–29.9 kg/m^2^	24.8	24.6	0.01
BMI 30.0–34.9 kg/m^2^	23.0	23.0	0.00
BMI 35.0–39.9 kg/m^2^	14.7	14.3	0.01
BMI ≥40 kg/m^2^	18.4	18.4	0.00
BMI < 18.5 kg/m^2^	1.3	1.2	0.01
Rheumatoid arthritis	6.2	5.8	0.02
Osteoarthritis	18.9	19.9	0.03
Headache	10.7	11.1	0.01
Diverticulitis	3.8	3.8	0.00
H. pylori treatment	0.4	0.6	0.02
Hypothyroidism	0.8	0.9	0.00
Solid tumor	9.5	10.2	0.02
Metastatic cancer	3.5	3.8	0.01
Major surgery	9.8	9.7	0.01
Varicose veins	1.4	1.3	0.01
**Comedications**			
Aspirin	22.7	24.0	0.03
P2Y12 platelet inhibitor	3.1	4.0	0.05
Nonsteroidal anti-inflammatory drug	31.8	30.8	0.02
Celecoxib	1.1	1.0	0.01
Angiotensin-converting enzyme inhibitor or receptor blocker	31.1	32.2	0.02
Beta-blocker	23.5	25.3	0.04
Diltiazem	1.8	2.0	0.01
Verapamil	0.9	1.1	0.02
Dihydropyridine calcium channel blocker	21.2	21.8	0.01
Loop diuretic	11.0	11.9	0.03
Thiazide	21.2	21.4	0.00
Digoxin	0.5	0.8	0.04
Statin	23.7	24.5	0.02
Other cholesterol medication	2.0	2.1	0.01
Metformin	11.7	11.5	0.01
Sulfonylurea or glinides	5.1	6.0	0.04
Thiazolidinediones	0.5	0.6	0.02
Dipeptidyl peptidase 4 inhibitors	1.5	1.4	0.01
Glucagon-like peptide-1 agonist	0.5	0.3	0.03
Insulin	7.8	8.9	0.04
Selective serotonin reuptake or serotonin-norepinephrine reuptake inhibitor	10.6	11.3	0.02
Other antidepressants	9.0	9.7	0.03
Proton pump inhibitors	21.4	22.6	0.03
Histamin-2 receptor antagonist	8.4	9.0	0.02
Systemic corticosteroids	17.8	18.8	0.03
Alpha-glucosidase inhibitor	0.1	0.0	0.04
Hypnotic medication	3.7	3.6	0.00
Sodium-glucose cotransporter-2 inhibitor	0.3	0.2	0.02

Baseline categorical data were reported as percentages and continuous data as medians with 25, 75% ranges. Endpoint incidence were reported as proportions (the number experiencing an event divided by total number of patients in the cohort). Weighted Cox proportional hazards regression analysis were performed using the **‘SURVEY’** package version 3.36 in **R**. Patients assigned weights at the extremes (<1st or > 99th percentile) were recalibrated to the corresponding threshold values prior to weighted regression analyses [14]. The proportional hazard assumption was tested based on Schoenfeld residuals and was found valid for all endpoints.

We performed sensitivity analyses in which an on-treatment approach (i.e., patients followed for up to 12-months or until endpoint occurrence, index OAC discontinuation [30-day permissible gap] or switch, end-of-EHR activity or through September 30, 2018) was utilized and differences in baseline characteristics between the rivaroxaban and warfarin cohorts were adjusted using IPTW based on propensity scores. An additional sensitivity analysis was performed in which differences in baseline characteristics between the rivaroxaban and warfarin cohorts were adjusted by propensity score matching calculated via multivariable logistic regression using **‘MatchIT’** in **R**. Each eligible rivaroxaban user was 1:1 propensity score matched using greedy nearest neighbor matching without replacement and a caliper = 0.25 standard deviations of the propensity score. For sensitivity analysis, Cox proportional hazards regression analysis was performed using IBM SPSS version 25.0 (IBM Corp, Armonk, NY).

The report for this analysis was written to comply with the Reporting of Studies Conducted using Observational Routinely-Collected Health Data (RECORD) statement [15].

## Results

In total, we identified 48,429 patients with a primary diagnosis for VTE with ≥1 provider visit in the 12-months prior to the index VTE event. Of these, 6158 were African Americans and 4939 were initiated on OAC with either rivaroxaban or warfarin within 7-days of the index event. The median age of included patients was 51 years, 56.4% o were female, 18.4% were morbidly obese (body mass index ≥40 kg/m^2^), 9.7% had a history of prior major surgery, 3.7 and 9.9% and had a history of/active metastatic cancer or a solid tumor, respectively, and nearly 1 in 5 patients had a PE ± DVT.

Following IPTW, patients were deemed well-balanced on all independent variables entered into the propensity-score model as demonstrated by absolute standardized differences between the rivaroxaban and warfarin users < 0.1. Upon Cox regression, rivaroxaban use (*N* = 2097) was not associated with a statistically significant difference in the incidence of the composite endpoint of recurrent VTE or major bleeding at 3-months (4.57% versus 4.58%, HR = 1.08, 95%CI = 0.82–1.42), 6-months (5.01% versus 5.84%, HR = 0.96, 95%CI = 0.75–1.24) or 12-months (5.82% versus 7.32%, HR = 0.93, 95%CI = 0.74–1.16) of follow-up (Table 2) compared to warfarin (*N* = 2842). No significant differences were observed between the cohorts for either of the components when evaluated separately at these same time points, nor were there significant differences in the incidence of ICH (HR = 0.66, 95%CI = 0.12–3.59 at 3-months; HR = 0.50, 95%CI = 0.10–2.50 at 6-months and HR = 0.76, 95%CI = 0.27–2.19 at 12-months), GI (HR = 1.16, 95%CI = 0.61–2.21 at 3-months; HR = 0.84, 95%CI = 0.47–1.51 at 6-months and HR = 0.80, 95%CI = 0.47–1.37 at 12-months) and GU bleeding (HR = 1.08, 95%CI = 0.30–3.93 at 3-months; HR = 0.80, 95%CI = 0.24–2.63 at 6-months and HR = 0.88, 95%CI = 0.29–2.63 at 12-months). Sensitivity analyses 1) employing propensity score matching (*N* = 2068 users of rivaroxaban or warfarin per group) and 2) based upon an on-treatment analysis approach both provided similar results to the base-case analysis for the composite endpoint, recurrent VTE and major bleeding alone at each timepoint (Tables 3 and 4).

**Table 2:** Event Incidence and Hazard Ratios with 95% Confidence Intervals for IPTW Analysis of the Rivaroxaban and Warfarin Cohorts Using an Intent-To-Treat Approach

	Rivaroxaban *N* = 2097 n (%)	Warfarin *N* = 2842 n (%)	HR (95%CI)
**3-Month**			
Composite of recurrent venous thromboembolism or major bleeding	96 (4.58)	130 (4.57)	1.08 (0.82–1.42)
Recurrent venous thromboembolism	74 (3.53)	96 (3.38)	1.07 (0.78–1.46)
Major bleeding	27 (1.29)	40 (1.41)	1.19 (0.72–1.97)
Intracranial hemorrhage	2 (0.10)	5 (0.18)	0.66 (0.12–3.59)
Gastrointestinal bleeding	17 (0.81)	24 (0.84)	1.16 (0.61–2.21)
Genitourinary bleeding	4 (0.19)	6 (0.21)	1.08 (0.30–3.93)
**6-Month**			
Composite of recurrent venous thromboembolism or major bleeding	105 (5.01)	166 (5.84)	0.96 (0.75–1.24)
Recurrent venous thromboembolism	81 (3.86)	115 (4.05)	1.01 (0.76–1.36)
Major bleeding	30 (1.43)	59 (2.08)	0.93 (0.59–1.47)
Intracranial hemorrhage	2 (0.10)	7 (0.25)	0.50 (0.10–2.50)
Gastrointestinal bleeding	18 (0.86)	37 (1.30)	0.84 (0.47–1.51)
Genitourinary bleeding	4 (0.19)	9 (0.32)	0.80 (0.24–2.63)
**12-Month**			
Composite of recurrent venous thromboembolism or major bleeding	122 (5.82)	208 (7.32)	0.93 (0.74–1.16)
Recurrent venous thromboembolism	89 (4.24)	140 (4.93)	0.95 (0.72–1.2)
Major bleeding	39 (1.86)	80 (2.81)	0.92 (0.62–1.36)
Intracranial hemorrhage	5 (0.24)	13 (0.46)	0.76 (0.27–2.19)
Gastrointestinal bleeding	21 (1.00)	47 (1.65)	0.80 (0.47–1.37)
Genitourinary bleeding	5 (0.24)	10 (0.35)	0.88 (0.29–2.63)

*CI* Confidence interval, *HR* Hazard ratio, *N* Number

**Table 3:** Characteristics of the 1:1 Propensity Score Matched (Sensitivity Analysis) Rivaroxaban and Warfarin Cohorts

	Rivaroxaban*N* = 2068%	Warfarin*N* = 2068%	Absolute Standardized Difference
**Demographics**			
Age, median (25, 75% range)	50 (39, 62)	51 (40, 64)	–
Age 18–49 years	46.32	48.21	0.04
Age 50–64 years	30.03	30.32	0.01
Age 65–74 years	13.10	12.28	0.03
Age 75–79 years	4.59	3.77	0.04
Age ≥ 80 years	5.95	5.42	0.02
Female sex	56.19	55.66	0.01
Pulmonary embolism (±deep vein thrombosis)	18.09	17.89	0.01
**Comorbidities**			
Chronic obstructive pulmonary disease	8.37	7.98	0.01
Asthma	13.25	13.10	0.00
Heart Failure	4.84	4.93	0.00
Hypertension	52.80	51.45	0.27
Ischemic stroke or transient ischemic attack	2.95	2.85	0.01
Diabetes	21.13	21.03	0.00
Dementia	2.18	2.03	0.01
Coronary artery disease	0.68	0.87	0.02
Carotid stenosis	0.63	0.53	0.01
Peripheral vascular disease	5.51	5.42	0.00
Myocardial infarction	5.08	4.59	0.02
Percutaneous coronary intervention	3.34	3.13	0.01
Coronary artery bypass grafting	1.93	2.18	0.02
Gastrointestinal bleed	0.24	0.29	0.01
Intracranial hemorrhage	0.00	0.00	NA
Acute kidney injury	10.06	9.38	0.02
Other kidney injury	0.24	0.24	0.00
Inflammatory bowel disease	0.77	0.82	0.01
eGFR > 90 mL/minute	55.42	58.32	0.06
eGFR 60–89 mL/minute	0.48	0.73	0.03
eGFR 30-59 mL/minute	31.09	28.97	0.05
eGFR 15–29 mL/minute	10.88	10.06	0.03
eGFR < 15 mL/minute	1.21	1.11	0.01
eGFR unknown	0.77	0.68	0.01
Liver disease	1.50	1.93	0.03
Coagulopathy	3.00	3.00	0.00
Gastroesophageal reflux disease	18.76	18.96	0.00
Anemia	24.13	23.55	0.01
Sleep apnea	10.20	10.64	0.01
Smoking	28.77	28.19	0.01
Hemorrhoids	2.22	2.37	0.01
Alcohol abuse	0.34	0.34	0.00
Anxiety	12.28	14.02	0.05
Depression	1.69	1.60	0.01
Psychosis	1.50	1.16	0.03
BMI < 18.5 kg/m^2^	1.60	1.74	0.01
BMI 18.5–24.9 kg/m^2^	15.43	14.46	0.03
BMI 25.0–29.9 kg/m^2^	24.71	25.29	0.01
BMI 30.0–34.9 kg/m^2^	23.26	23.79	0.01
BMI 35.0–39.9 kg/m^2^	14.70	15.47	0.02
BMI ≥40 kg/m^2^	19.15	17.65	0.04
BMI unknown	1.16	1.60	0.03
Rheumatoid arthritis	5.80	6.53	0.03
Osteoarthritis	18.86	18.23	0.02
Headache	10.15	10.59	0.01
Diverticulitis	3.72	3.77	0.00
H. pylori treatment	0.39	0.34	0.01
Hypothyroidism	0.87	0.87	0.00
Solid tumor	9.72	8.90	0.03
Metastatic cancer	3.72	3.29	0.02
Major surgery	10.11	10.01	0.00
Varicose veins	1.26	1.35	0.01
**Comedications**			
Aspirin	22.10	21.47	0.02
P2Y12 platelet inhibitor	2.90	2.80	0.01
Nonsteroidal anti-inflammatory drug	31.33	33.95	0.05
Celecoxib	1.02	1.35	0.03
Angiotensin-converting enzyme inhibitor or receptor blocker	30.66	29.21	0.03
Beta-blocker	22.44	21.52	0.02
Diltiazem	1.55	1.69	0.01
Verapamil	0.87	0.87	0.00
Dihydropyridine calcium channel blocker	20.31	19.44	0.02
Loop diuretic	10.69	9.91	0.03
Thiazide	21.08	20.45	0.02
Digoxin	0.44	0.39	0.01
Statin	23.02	21.66	0.03
Other cholesterol medication	2.13	1.98	0.01
Metformin	11.41	11.17	0.01
Sulfonylurea or glinides	4.64	4.30	0.02
Thiazolidinediones	0.34	0.53	0.03
Dipeptidyl peptidase 4 inhibitors	1.35	1.64	0.02
Glucagon-like peptide-1 agonist	0.29	0.44	0.02
Insulin	7.54	7.06	0.02
Selective serotonin reuptake or serotonin-norepinephrine reuptake inhibitor	10.88	10.74	0.00
Other antidepressants	8.95	8.80	0.01
Proton pump inhibitors	21.23	21.03	0.00
Histamin-2 receptor antagonist	9.14	8.46	0.02
Systemic corticosteroids	18.13	18.09	0.00
Alpha-glucosidase inhibitor	0.00	0.00	0.00
Hypnotic medication	3.68	4.01	0.02
Sodium-glucose cotransporter-2 inhibitor	0.15	0.19	0.01

**Table 4:** Results of Sensitivity Analyses

	1:1 Propensity Score Matching	On-Treatment Approach
**3-Month**		
Composite of recurrent venous thromboembolism or major bleeding	1.10 (0.82–1.46)	1.10 (0.83–1.45)
Recurrent venous thromboembolism	1.08 (0.78–1.50)	1.11 (0.81–1.52)
Major bleeding	1.28 (0.73–2.25)	1.17 (0.69–1.98)
Intracranial hemorrhage	1.04 (0.15–7.37)	0.65 (0.12–3.47)
Gastrointestinal bleeding	1.11 (0.56–2.19)	1.08 (0.55–2.13)
Genitourinary bleeding	1.39 (0.31–6.21)	1.05 (0.29–3.75)
**6-Month**		
Composite of recurrent venous thromboembolism or major bleeding	1.00 (0.767–1.31)	1.05 (0.81–1.37)
Recurrent venous thromboembolism	1.04 (0.76–1.41)	1.12 (0.83–1.53)
Major bleeding	1.02 (0.62–1.69)	1.01 (0.62–1.66)
Intracranial hemorrhage	0.70 (0.12–4.20)	0.65 (0.12–3.47)
Gastrointestinal bleeding	0.80 (0.43–1.47)	0.94 (0.50–1.78)
Genitourinary bleeding	1.39 (0.31–6.21)	0.85 (0.25–2.84)
**12-Month**		
Composite of recurrent venous thromboembolism or major bleeding	0.98 (0.76–1.25)	1.04 (0.80–1.34)
Recurrent venous thromboembolism	1.00 (0.75–1.34)	1.10 (0.82–1.47)
Major bleeding	0.99 (0.64–1.5)	0.98 (0.61–1.57)
Intracranial hemorrhage	0.94 (0.29–3.10)	0.82 (0.21–3.30)
Gastrointestinal bleeding	0.84 (0.47–1.48)	0.88 (0.47–1.65)
Genitourinary bleeding	1.34 (0.36–5.00)	1.03 (0.33–3.24)

## Discussion

This EHR-based study evaluated African American patients experiencing a VTE treated with rivaroxaban or warfarin in routine practice. Our analysis suggested there was no significant difference in the incidence of the composite endpoint of recurrent VTE or major bleeding between the treatment groups after 3-, 6- or 12-months of follow up. No significant differences were observed between the cohorts for either of the components when evaluated separately at these same time points, nor were there significant differences in the incidence of ICH, GI or GU bleeding. Our conclusions were also similar when an on-treatment approach and propensity score matching were utilized.

African American patients have been under-enrolled in RCTs evaluating NOACs for the treatment of VTE [7–10]. Moreover, no sub-analyses of an RCT has reported on the efficacy and/or safety of NOACs in a cohort of African American patients. Consequently, our present analysis provides important new data to aid in clinical decision-making. The findings of our study were generally consistent with those of the pooled EINSTEIN trial analysis which included a small portion (2.6%) of black patients and the prospective, nonrandomized XALIA registry study [5, 9, 16]. In the pooled EINSTEIN trial analysis, rivaroxaban (*n* = 4151) was found to be non-inferior to enoxaparin/vitamin K antagonist (VKA) (*n* = 4131) for the endpoint of recurrent VTE with a 2.1 and 2.3% incidence, respectively (HR = 0.89; 95%CI = 0.66–1.19). These results were echoed in XALIA which found no significant difference in recurrent VTE risk between rivaroxaban (*n* = 2619) (1.4%) and standard-of-care management (typically parenteral bridging to a VKA) (*n* = 2149) (2.3%) of acute DVT (±PE) in routine practice (propensity score-adjusted HR = 0.91; 95%CI = 0·54–1·54). While reductions in the risk of major bleeding was observed with rivaroxaban (1.0%) compared to enoxaparin/VKA (1.7%) in the EINSTEIN clinical trial program (HR = 0.54; 95%CI = 0.37–0.79), no significant difference was observed in XALIA for rivaroxaban (0.8%) versus standard-of-care (2.1%) management (propensity score-adjusted HR = 0.77; 95%CI = 0.40–1.50).

This study has limitations worthy of discussion. First, multiple biases including misclassification, sampling and confounding bias are always important limitations in non-randomized, retrospective studies that may impact their internal validity [17]. We attempted to reduce the probability of misclassification bias by using validated coding schema to identify comorbidities and endpoints (when possible). Our methodology appeared to be effective given we reported recurrent VTE rates just slightly above previously published studies [9, 16, 18] (which was anticipated as black patients have been hypothesized to be at a higher risk of thrombosis compared to other races [1–5]) as well as a similar incidence of major bleeding (range in prior studies: 0.77–2.1%) [9, 16, 18]. Second, we used EHR data for US patients [11]; and therefore, our results are most generalizable to a US population. Third, we did not calculate time in therapeutic international normalized ratio (INR) range for warfarin patients. While INR control is somewhat predictive of the effectiveness and safety of vitamin K antagonists, it is well known that VTE patients treated with warfarin in routine US clinical practice spend only ~ 56% of their time during the first 3 months of treatment (when recurrent VTE is most likely) in the therapeutic INR range [19, 20] and African American patients may have among the poorest INR control [21]. Given the Optum EHR data used for this study covers patients throughout the US regardless of insurer (or no insurance), it is likely patients included in this study experienced at least similar suboptimal INR control. Fourth, results of the on-treatment analysis which was performed to supplement our intention-to-treat analysis should be interpreted with caution as it is unclear how accurately EHRs maintain up-to-date patient medication profiles. Moreover, an EHR entry to initiate an OAC does neither guarantee a patient took the medication nor (as in claim data sets) assures patients even picked up their prescription at the pharmacy. Finally, absent randomization in a study, residual confounding cannot be fully excluded due to the possibility of confounding from unobserved or unmeasured covariates [11].

## Conclusions

In conclusion, our EHR-based study suggests rivaroxaban is no worse than warfarin at preventing recurrent VTE with no increased risk of major bleeds in African Americans. Our findings are similar to those found in the EINSTEIN clinical trials and consistent with the results from the XALIA study. Additional real-world analyses with an increased sample size to evaluate the effectiveness and safety of rivaroxaban for treatment and prevention of VTE in African Americans are warranted.

## Data Availability

Data used in this study were obtained from Optum under a license to Janssen Scientific Affairs LLC (and provided to Dr. Coleman under a third-party agreement) and are not publicly available.

## Abbreviations

CI: Confidence intervals
DVT: Deep vein thrombosis
EHR: Electronic health record
HR: Hazard ratio
ICH: Intracranial hemorrhage
IPTW: Inverse probability-of-treatment weighting
GI: Gastrointestinal
GU: Genitourinary
NOACs: Non-vitamin K oral anticoagulants
OAC: Oral anticoagulant
PE: Pulmonary embolism
RCTs: Randomized controlled trials
RECORD: Reporting of studies conducting using observational routinely-collected health data
US: United States
VTE: Venous thromboembolism

## References

[1] White RH, Zhou H, Romano PS (1998). Incidence of idiopathic deep venous thrombosis and secondary thromboembolism among ethnic groups in California. Ann Intern Med.

[2] White RH, Zhou H, Murin S, Harvey D (2005). Effect of ethnicity and gender on the incidence of venous thromboembolism in a diverse population in California in 1996. Thromb Haemost.

[3] Schneider D, Lilienfeld DE, Im W (2006). The epidemiology of pulmonary embolism: racial contrasts in incidence and in-hospital case fatality. J Natl Med Assoc.

[4] Dowling NF, Austin H, Dilley A, Whitsett C, Evatt BL, Hooper WC (2003). The epidemiology of venous thromboembolism in Caucasians and African-Americans: the GATE study. J Thromb Haemost.

[5] Jackson LR, Peterson ED, Okeagu E, Thomas K (2015). Review of race/ethnicity in non vitamin K antagonist oral anticoagulants clinical trials. J Thromb Thrombolysis.

[6] Di Nisio M, Ageno W, Rutjes AW, Pap AF, Büller HR (2016). Risk of major bleeding in patients with venous thromboembolism treated with rivaroxaban or with heparin and vitamin K antagonists. Thromb Haemost.

[7] Agnelli G, Buller HR, Cohen A (2013). Oral apixaban for the treatment of acute venous thromboembolism. N Engl J Med.

[8] Buller HR, Decousus H, Grosso MA (2014). Edoxaban versus warfarin for the treatment of symptomatic venous thromboembolism. N Engl J Med.

[9] Prins MH, Lensing AW, Bauersachs R (2013). Oral rivaroxaban versus standard therapy for the treatment of symptomatic venous thromboembolism: a pooled analysis of the EINSTEIN-DVT and PE randomized studies. Thromb J.

[10] Schulman S, Kearson C, Kakkar AK (2009). Dabigatran versus warfarin in the treatment of acute venous thromboembolism. N Engl J Med.

[11] Optum. Optum EHR Offering. Optum Inc, 2018. Available at: https://www.optum.com/campaign/ls/data-new-era-of-visibility/download.html (Last accessed on July 28, 2019).

[12] White RH, Garcia M, Sadeghi B (2010). Evaluation of the predictive value of ICD-9-CM coded administrative data for venous thromboembolism in the United States. Thromb Res.

[13] Cunningham A, Stein CM, Chung CP, Daugherty JR, Smalley WE, Ray WA (2011). An automated database case definition for serious bleeding related to oral anticoagulant use. Pharmacoepidemiol Drug Saf.

[14] Austin PC (2011). An introduction to propensity score methods for reducing the effects of confounding in observational studies. Multivariate Behav Res.

[15] Benchimol EI, Smeeth L, Guttmann A, Harron K, Moher D, Petersen I, Sørensen HT, von Elm E, Langan SM (2015). RECORD Working Committee. The REporting of studies Conducted using Observational Routinely-collected health Data (RECORD) statement. PLoS Med.

[16] Ageno W, Mantovani LG, Haas S (2016). Safety and effectiveness of oral rivaroxaban versus standard anticoagulation for the treatment of symptomatic deep-vein thrombosis (XALIA): an international prospective, non-interventional study. Lancet Haematol.

[17] Gandhi SK, Salmon W, Kong SX, Zhao SZ (1999). Administrative databases and outcomes assessment: an overview of issues and potential utility. J Manag Care Spec Pharm.

[18] Coleman CI, Turpie AG, Bunz TJ, Beyer-Westendorf J. Effectiveness and safety of rivaroxaban versus warfarin in patients with provoked venous thromboembolism. J Thromb Thrombolysis. 2018;46:339–45. 10.1007/s11239-018-1695-1 29881958

[19] Erkens PM, ten Cate H, Büller HR, Prins MH (2012). Benchmark for time in therapeutic range in venous thromboembolism: a systematic review and meta-analysis. PLoS One.

[20] Limone BL, Hernandez AV, Michalak D, Bookhart BK, Coleman CI (2013). Timing of recurrent venous thromboembolism early after the index event: a meta-analysis of randomized controlled trials. Thromb Res.

[21] Yong C, Xu X, Than C, Ullal A, Schmitt S, Azarbal F, Heidenreich P, Turakhia M (2013). Racial disparities in warfarin time in INR therapeutic range in patients with atrial fibrillation: findings from the TREAT-AF study. Circulation.

